# The implications for urological malignancies of non-coding RNAs in the the tumor microenvironment

**DOI:** 10.1016/j.csbj.2023.12.016

**Published:** 2023-12-20

**Authors:** Shijin Wang, Xiaochen Qi, Dequan Liu, Deqian Xie, Bowen Jiang, Jin Wang, Xiaoxi Wang, Guangzhen Wu

**Affiliations:** a Department of Urology, The First Affiliated Hospital of Dalian Medical University, Dalian 116011, Liaoning, China; b Department of Clinical Laboratory Medicine, The First Affiliated Hospital of Dalian Medical University, Dalian 116011, Liaoning, China

**Keywords:** The tumor microenvironment, Non-coding RNA, Urological malignancies

## Abstract

Urological malignancies are a major global health issue because of their complexity and the wide range of ways they affect patients. There's a growing need for in-depth research into these cancers, especially at the molecular level. Recent studies have highlighted the importance of non-coding RNAs (ncRNAs) – these don't code for proteins but are crucial in controlling genes – and the tumor microenvironment (TME), which is no longer seen as just a background factor but as an active player in cancer progression. Understanding how ncRNAs and the TME interact is key for finding new ways to diagnose and predict outcomes in urological cancers, and for developing new treatments. This article reviews the basic features of ncRNAs and goes into detail about their various roles in the TME, focusing specifically on how different ncRNAs function and act in urological malignancies.

## Graphical Abstract

**Figure:**
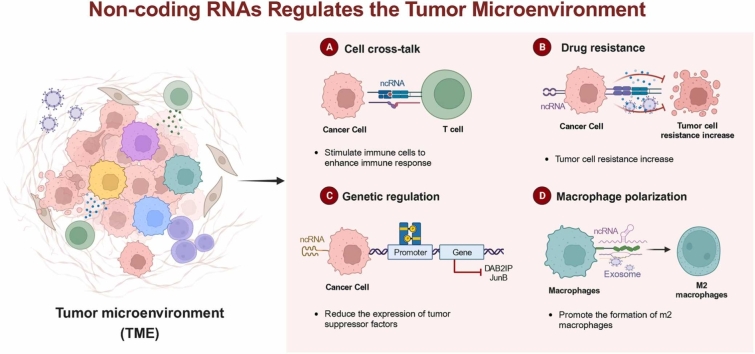

## 1 Introduction

Urological malignancies constitute a substantial threat to worldwide health [1], [2]. These malignancies cover a wide range of diseases, including cancers of the prostate, bladder, kidney, and testicle, among others. It is imperative to underscore the severity of prostate cancer, holding the title of the second most common cancer in males across the globe. The Global Cancer Statistics 2020 reported an incredible 1.4 million fresh cases and a grim total of 375,000 fatalities in just the year 2020[3]. According to estimates, 151,297 people in Europe were estimated to have bladder cancer in 2012, with age-standardized incidence rates (per 100,000 people) of 17.7 for men and 3.5 for women[4]. Age, lifestyle, including smoking and food, and genetic liability are some risk factors affecting these malignancies [5], [6]. Despite a wide range of available therapies, including targeted therapy, radiation therapy, chemotherapy, immunotherapy, and surgical intervention4, the incidence and mortality toll continue to rise. This pathetic reality highlights the essential urgency for treatments of higher efficacy.

The TME emerges as a critical factor in the advancement of cancer. This dynamic and complex ecosystem is composed of diverse cellular constituents, such as cancer cells, immune cells, fibroblasts, and endothelial cells, all embedded within the elaborate network of the extracellular matrix [7], [8], [9]. The architecture and operational dynamics of the TME are not static but vary according to the type and stage of the tumor, as well as the patient’s unique characteristics. The TME exerts a significant influence on every stage of tumor development, encompassing carcinogenesis, tumor growth, angiogenesis, invasion, and metastasis [7], [8], [10], [11].

NcRNAs represent a varied group of RNA molecules that, despite not being involved in protein synthesis, are pivotal in various cellular processes. NcRNAs are broadly classified into small ncRNAs and long ncRNAs (lncRNAs). Small ncRNAs, typically under 200 nucleotides, include microRNAs (miRNAs), small interfering RNAs (siRNAs), and Piwi-interacting RNAs (piRNAs). These play significant roles in biological functions such as cellular growth, differentiation, and proliferation [12], [13]. Advancements in High-Throughput Sequencing (HTS) and bioinformatics tools have greatly enhanced our comprehension of ncRNA functions and their significant roles in disease pathogenesis. MicroRNAs, for example, are vital in post-transcriptional gene regulation. They achieve this by binding to the 3′ untranslated region (UTR) of target mRNAs, leading to either inhibition of translation or mRNA degradation, thus influencing various biological processes [14], [15]. Contrastingly, lncRNAs, predominantly longer than 200 nucleotides, have a broad interaction spectrum involving DNA, RNA, and proteins. This interaction facilitates diverse cellular activities ranging from gene expression and chromatin remodeling to molecular trafficking[16], [17]. Besides miRNAs and lncRNAs, other ncRNAs like circular RNA (circRNAs) and transfer RNAs (tRNAs) also contribute uniquely to cellular functions. CircRNAs, notable for their circular configuration, are implicated in gene regulation and are frequently dysregulated in diseases, including cancer[18].

In this review, we have amalgamated the latest insights from advanced technologies, such as HTS and bioinformatics, to enhance our understanding of ncRNAs in tumor initiation, progression, and resistance to treatment. Additionally, this article delves into the complex interplay between ncRNAs and the TME specifically in urological malignancies. This exploration provides valuable insights and crucial information, which could be instrumental in developing future therapeutic strategies.

## 2 Overview of NcRNAs

NcRNAs form a diverse group of RNA molecules that play significant roles in cellular functions, despite not being involved in protein encoding [19]. MiRNAs, a subset of small ncRNAs approximately 22 nucleotides in length, act as key regulators of gene expression at the post-transcriptional level. They exert their function by binding to the 3′ UTRs of their target mRNAs, leading to either mRNA degradation or translational inhibition [20]. The biogenesis of miRNAs commences with the transcription of a primary miRNA (pri-miRNA), which is then processed into a precursor miRNA (pre-miRNA) by the Drosha-DGCR8 complex in the nucleus. Subsequently, the pre-miRNA is transported to the cytoplasm and further refined into its mature form by the enzyme Dicer. Upon maturation, the miRNA integrates into the RNA-induced silencing complex (RISC), targeting specific mRNA molecules for regulation [21]. LncRNAs, exceeding 200 nucleotides in length, are involved in various biological processes, including chromatin remodeling, transcriptional, and post-transcriptional regulation [22], [23]. LncRNAs are transcribed and interact with DNA, RNA, and proteins to execute their diverse functions. Notably, some lncRNAs can also serve as precursors for small ncRNAs [21]. CircRNAs, characterized by their closed-loop structure resulting from back-splicing, form another unique class of ncRNAs. Their functions in cellular processes are varied, such as acting as miRNA sponges, interacting with RBPss, and in certain instances, encoding proteins [24], [25]. Understanding the general biological roles of ncRNAs is fundamental before delving into their specific functions within the TME of urological malignancies.

### 2.1 NcRNAs: the invisible movers of genetic interactions

NcRNAs, including miRNAs, lncRNAs, and circRNAs, play crucial roles in cellular processes such as gene regulation, cell differentiation, and development. MiRNAs, for instance, regulate gene expression by destabilizing messenger RNAs (mRNAs) and inhibiting their translation, thus impacting a range of cellular functions [26]. An illustrative example is the overexpression of MicroRNA-361–3p, leading to decreased SH2B1 levels, which in turn inhibits the proliferation and spread of Non-Small Cell Lung Cancer cells[27]. Additionally, miRNAs are instrumental in cellular development, particularly in regulating macrophage activation. They modulate the effects of Th2 cell cytokines interleukin-4 (IL-4) and IL-13 on macrophage biology, biosynthesis, and environmental response, underscoring their importance in immune cell development and reactivity [28].

LncRNAs, another type of ncRNA, regulate gene expression at various stages, including chromatin modification, transcription, and post-transcriptional processing. A prominent example is linc-MD1 in mouse and human myoblasts, which acts as a 'sponge' for miR-133 and miR-135, thereby regulating the transcription factors MAML1 and MEF2C crucial for muscle-specific gene expression and muscle cell differentiation[29]. LncRNAs such as HOTAIR function as scaffolds for protein complexes, like the combination of PRC2 and the LSD1/CoREST/REST complex, to orchestrate gene silencing [30]. Additionally, certain lncRNAs function as molecular decoys; for instance, lncRNA GAS5 inhibits the binding of the glucocorticoid receptor to target gene promoters [31]. In the realm of cellular differentiation, the lncRNA Braveheart (Bvht) is critical for cardiovascular lineage commitment in mouse embryonic stem cells, mediating epigenetic changes essential for activating cardiovascular genes through its interaction with the transcription factor SUZ12, a component of PRC2 [32]. Furthermore, certain lncRNAs exhibit enhancer-like functions, activating key regulators of development and differentiation in human cell lines [33]. CircRNAs, too, play significant roles in gene regulation and cell differentiation. For instance, ciRS-7 functions as a miRNA sponge, regulating gene expression by sequestering miR-7[34]. The circRNA hsa-circ-0074834 promotes osteogenic differentiation of bone marrow stem cells by regulating ZEB1 and VEGF expression via the downregulation of miR-942–5p, highlighting circRNAs' roles in gene regulation and development [35]. Interestingly, some circRNAs, like circ-ZNF609, have been found to possess protein-coding potential, expanding our understanding of the functional diversity of circRNAs in cellular processes [36].

In summary, ncRNAs are integral in directing gene expression and various cellular processes, and their dysregulation is often associated with diseases, including cancer, making them potential targets for therapeutic interventions (Table 1).

**Table 1:** The Timeline of ncRNA Studies in Cancer Research.

Time Period	Years	Methods	ncRNA Types	Cancer Type	Species	Key Advancements	Ref
Early Research	2000	Northern Blotting	PCGEM1	Pca	Human	PCGEM1 is notably overexpressed in prostate cancer and lacks the capacity to encode proteins. Its heightened expression promotes tumor cell proliferation and survival	[37]
	2002	Northern Blotting and miR-Array	miR15 and *miR16*	CLL	Human	miR15 and miR16 on chromosome 13q14 are missing in B-CLL cases	[38], [39]
Early Research	2003	Northern Blotting	MALAT-1	NSCLC	Human	High MALAT-1 expression was associated with poor survival in early-stage NSCLC	[40]
	2004	Northern Blotting	let-7	NSCLC	Human	Overexpression of let-7 in lung adenocarcinoma cell line A549 inhibited cancer cell growth	[41]
	2005	Northern Blotting	miR-17-92	LCA	Human	The copy number of the miR-17-92 gene is increased, and its overexpression promotes lung cancer cell growth	[42]
	2005	miR-Array and Northern Blotting	miR-21	GBM	Human	Overexpression of miR-21 in glioblastoma contributes to the malignant phenotype by blocking apoptosis-related genes	[43]
	2006	miR-Array	miRNA-372 and miRNA-373	TGCT	Human and nude mice	identification of miR-372 and miR-373 as potential oncogenes that can promote proliferation and tumorigenesis by interfering with the p53 pathway	[44]
	2008	Northern Blotting and miR-Array	H19	COAD and LIHC	Mice	H19 locus functions as a tumor suppressor	[45]
Advent of RNA-seq	2011	RNA-Seq and RIP	PCAT-1	Pca	Human	PCAT-1, is notably overexpressed in metastatic prostate cancer, where it influences cell proliferation and is regulated by the EZH2 protein	[46]
	2016	RNA-seq and qRT-PCR	F-circRNA	leukemia	Human and nude mice	Chromosomal translocations produce f-circrna, which facilitates cell transformation, promotes cell viability and confers therapeutic resistance	[47]
	2016	RNA-Seq and ChIP-Seq	miR-515-5p	BC and NSCLC	Human and nude mice	miR-515-5p plays a significant role in inhibiting cell migration by directly down-regulating MARK4 expression in two different cancer types (breast and lung)	[48]
	2017	RNA-seq	CircCCDC66	CRC	Human and nude mice	Knockdown of circCCDC66 significantly reduced cell number, suppressed cell proliferation, migration, and invasion in HCT116 and HT-29 cell lines	[49]
	2017	RNA-seq,RIP and ChIP	CCAT1	ESCC	Human and nude mice	By sequestering miR-7, CCAT1 facilitates increased expression of HOXB13, thus promoting cell growth and migration	[50]
Advent of RNA-seq	2017	RNA-seq,Cell-cycle analysis and qRT-PCR	miR-6883 Family	CRC	Human	miR-6883-5p and miR-149 target CDK4/6, which disrupts FOXM1 phosphorylation and activation	[51]
	2018	RNA-seq and qRT-PCR	circDDX17	CRC	Human	circDDX17 is a tumor suppressor in colorectal cancer	[52]
	2018	RNA-seq,qRT-PCR and RIP	MIR22HG	NSCLC	Human	MIR22HG acts as a tumor suppressor in lung cancer, influencing cell survival, death signals, and oncogene regulation, particularly targeting YBX1, MET, and p21	[53]
	2018	RNA-seq and microRNA-seq	miRNAs	OSCC	Human and nude mice	miR-503-5p, miR-450b-5p, miR-27a-3p, miR-181a-5p, and miR-183-5p, can synergistically suppress the expression of RORA, a tumor suppressor	[54]
	2019	RNA-seq,RIP,qRT-PCR,ChIP and FISH	LINC01123	NSCLC	Human and nude mice	LINC01123 promotes cell proliferation, aerobic glycolysis, and metabolic reprogramming via c-Myc/miR-199a-5p axis	[55]
	2019	RNA-seq,exosomal communication and RIP	circNRIP1	GC	Human and nude mice	circNRIP1 functions as a miRNA sponge for miR-149-5p, enhancing malignant characteristics in gastric cancer via the AKT1/mTOR pathway.	[56]
	2019	RNA-seq,dual-luciferase reporter assays and ChIP	LINC00673	BC	Human and nude mice	LINC00673, regulated by YY1, enhances cell proliferation and tumor growth in breast cancer by modulating the MARK4/Hippo signaling pathway and interacting with miR-515-5p	[57]
	2019	RIP-seq, RIP-qPCR and FISH	*CDR1as*	GBM	Human and nude mice	CDR1as disrupts the p53/MDM2 complex by interacting with the DNA-binding domain of p53, subsequently inhibiting tumor growth	[58]
	2020	RNA-seq	miR-181b-3p	BC	Human and cell lines	FTO/miR-181b-3p/ARL5B signaling pathway as a target for therapeutic intervention in her2-positive breast cancer	[59]
	2020	RNA-seq,RNA pull-down and RIP	lncRNA XIST	CRC	Human and nude mice	METTL14 promotes m6A methylation in XIST, which inhibits proliferation and invasion in CRC	[60]
	2021	RNA-seq	miR-155-5p and miR-221-5p	PDAC	Human and nude mice	M2 macrophage-derived exosomes carrying miR-155-5p and miR-221-5p suppress E2F2 expression in endothelial cells, thereby promoting angiogenesis and contributing to the progression of PDAC	[61]
Advent of RNA-seq	2021	RNA-seq,RNA pull-down and RIP	LCAT3	LUAD	Humanand nude mice	LCAT3 recruits FUBP1 to the MYC far upstream element (FUSE) sequence, thereby activating MYC transcription	[62]
	2022	RNA-seq,RNA pull-down, RIP and FISH	TLNC1	LC	Human and nude mice	The interaction between TLNC1 and TPR promotes cytoplasmic translocation of the p53 protein, thereby inhibiting its transcriptional activity	[63]
	2022	RNA-Seq and (ceRNA) network analysis	LINC00680	ESCC	Human and nude mice	LINC00680 drives the malignant phenotype of ESCC by regulating PAK6 through sponging miR-423-5p	[64]
	2022	RIP-seq,RNA-seq,RNA-FISH and RNA pulldown	CircARID1A	GC	Human and nude mice	By stabilizing SLC7A5 and forming a ternary complex with IGF2BP3, circARID1A enhances GC cell growth	[65]
	2023	RNA-seq,RNA pull-down,FISH and RIP	SLCO4A1-AS	NSCLC	Humanand nude mice	SLCO4A1-AS1 inhibits lung cancer progression by disrupting the binding of TOX4 to the NTSR1 promoter	[66]
	2023	RNA-seq,RNA pull-down and RIP	SP100-AS1	CRC	Human and nude mice	SP100-AS1 regulates autophagy and radioresistance in CRC cells by sparing miR-622 and stabilizing ATG3	[67]
	2023	RNA-seq,RIP and RNA pull-down	LncGMDS-AS1	CRC	Human and nude mice	Activation of Jak-STAT3 and Wnt signaling pathways by GMDS-AS1 drives the malignant behavior of CRC	[68]
CRISPR Era	2017	CRISPR/Cas9	Linc-RoR	BC	Human	Linc-RoR promotes estrogen-dependent growth and resistance to tamoxifen by regulating the MAPK/ERK signaling pathway and DUSP7 stability	[69]
	2018	CRISPR/Cas9 and RIP	CYTOR	CRC	Human and nude mice	CYTOR forms a complex with NCL and Sam68. This CYTOR-NCL-Sam68 complex activates the NF-κB signaling pathway, contributing to the progression of CRC	[70]
	2019	CRISPR/Cas9	TTTY15	PCa	Human and nude mice	TTTY15 is upregulated in most PCa samples and promotes cancer progression by acting as a ceRNA for let-7	[71]
	2020	CRISPR/Cas9,RIP and RNA pull-down	LIN28B-AS1	HCC	Human and nude mice	The overexpression of LIN28B-AS1 leads to the upregulation of IGF2BP1, thereby promoting the progression of HCC cells	[72]
CRISPR Era	2023	CRISPR/Cas9 and FISH	NEAT1	MM	Human	The overexpression of NEAT1 provides oncogenic and prosurvival benefits to MM cells, particularly under stressors like nutrient deprivation or hypoxic conditions	[73]
Integration of Multi-method	2015	Northern blotting,ChIP and RNA pull-down	HOTAIR	GBC	Human and cell lines	HOTAIR functions as an oncogene activated by c-Myc, which negatively regulates miRNA-130a expression, contributing to the malignant progression of gallbladder cancers	[74]
	2017	RNA-seq,RNA-FISH and Northern Blotting	Neat1	PDAC	Humanand nude mice	Neat1, a gene regulated by p53, plays a crucial role in inhibiting cellular transformation	[75]
	2017	RIP-seq,mRNA-seq,Northern and Blotting	*LAST*	Not explicitly stated	Human and nude mice	LAST acts as a crucial regulator of CCND1 mRNA stability, promoting cell proliferation and facilitating the G1/S phase transition in the cell cycle	[76]
	2018	Transcriptome sequencing,RIP and ChIP	HOXC-AS3	GC	Human and cell lines	Interaction of HOXC-AS3 with YBX1 was found to regulate GC cell proliferation and migration	[77]
	2018	RNA-seq，RNC-seq, FISH and CRISPR/Cas9	CircPINTexon2	GBM	Human and cell lines	circPINTexon2 encodes the amino acid peptide PINT87aa, which interacts with the polymerase-associated factor complex (PAF1c) and represses transcriptional elongation of oncogenes.	[78]
	2018	RNA-seq,ChIP and CRISPR/Cas9	miR-100 and miR-125b	PDAC	Human and nude mice	Silencing miR-100 or miR-125b promotes MET and reduces cell migration and metastatic spread in vivo	[79]
	2020	CRISPR-CAS9,ChIP-qPCR and RIP	CircSOD2	HCC	Human and nude mice	circSOD2 functions as a miR-502-5p sponge, promoting the malignant progression of HCC by activating the JAK2/STAT3 pathway	[80]
	2021	CRISPR/Cas9,RNA-seq and FISH	lnc-HLX-2-7	MB	Human and nude mice	lnc-HLX-2-7 affects the metabolic status of MB by regulating HLX expression	[81]
	2022	CRISPR/Cas9,RIP,RNA-seq and ChIP-seq	*SNHG1*	NB	Human	SNHG1 interacts with histone deacetylases HDAC1 and HDAC2, influencing the chromatin state of genes and playing a crucial role in neuroblastoma	[82]
Integration of Multi-method	2023	CRISPR/Cas9 and RNA-seq	LINC00673	PDAC	Human and nude mice	LINC00673 encodes the protein RASON. This protein directly binds to KRAS and inhibits GAP-induced GTP hydrolysis, thereby maintaining KRAS in an active, GTP-bound state	[83]
	2023	CRISPR/Cas9,RNA-seq,RNA pull-down,RIP and FISH	*RIME*	ESCC	Human and nude mice	RIME modulates various immune checkpoint molecules, including PD-L1 and IDO-1, to facilitate immune escape by employing the RIME-MLL1-H3K4me3 axis	[84]

Breast Cancer: BC, Colorectal cancer: CRC, Colon adenocarcinoma: COAD, Esophageal squamous cell carcinoma: ESCC, Gallbladder cancer: GBC, Gastric cancer: GC, Glioblastoma: GBM, Hepatocellular carcinoma: HCC, Lung adenocarcinomas:LUAD, Lung Cancers: LCA, liver cancer: LC, Medulloblastoma:MB, Multiple myeloma:MM, Neuroblastoma:NB, Non-small cell lung cancer: NSCLC, Oral squamous cell carcinoma: OSCC, Pancreatic ductal adenocarcinoma:PDAC, Prostate cancer: PCa, Testicular germ cell tumor: TGCT

### 2.2 The impact of genetic variations on ncRNA networks and disease development

Transcriptome-Wide Association Studies (TWAS) have played a crucial role in uncovering genetic variations that are linked with health conditions, including aspects of cancer risk, prognosis, and drug response [85]. These studies draw particular attention to the impact of single nucleotide polymorphisms (SNPs) and copy number variations (CNVs), which are found to significantly influence the complex networks of ncRNAs.

SNPs within ncRNAs are known to influence disease development through several pathways. A notable mechanism involves the alteration of splicing models, where SNPs impact the splicing of lncRNA transcripts. This process results in the production of various isoforms with differing binding affinities, subsequently affecting the regulation of genes and cellular pathways. For instance, the lncRNA lnc13, implicated in inflammatory diseases, can be influenced by SNP (rs917997). This variation can lead to an increase in the levels of lnc13-regulated genes, heightening the risk of diseases like coeliac [86]. Similarly, an increase in the expression of lncRNA BCLET, associated with a reduced risk of bladder cancer, can be attributed to SNP rs558814, potentially improving survival rates [87]. In addition, SNPs can alter ncRNA expression levels, influencing susceptibility to various diseases. An example of this is SNP rs2839698 in lncRNA H19, linked to a decreased risk of non-muscle-invasive bladder cancer [88]. Another SNP, rs67311347, enhances the expression of ENTPD3-AS1, which acts protectively in renal cell carcinoma (RCC) by inhibiting cell proliferation via the miR-155–5p/HIF-1α pathway [89]. The SNP rs11672691, situated in a region that acts as a promoter for the short isoform of PCAT19 and as an enhancer for the long isoform, along with its linked SNP rs887391, reduces the binding affinity of transcription factors such as NKX3.1 and YY1. This reduced binding leads to a decrease in the expression of the tumor-suppressing PCAT19-short and an increase in the oncogenic PCAT19-long, promoting cell proliferation and tumor growth through interaction with HNRNPAB [90]. Moreover, SNPs located within lncRNA genes can disrupt their normal functions, contributing to disease by altering critical transcriptional regulatory networks. For example, SNP rs6983267, associated with the lncRNA CCAT1-L, can affect the expression of the MYC oncogene, a significant factor in many cancers [91], [92].

Moreover, SNPs enriched within lncRNAs can also affect their interactions with RNA-binding proteins (RBPs), essential for regulating gene expression [93], [94]. In bladder cancer, the C allele of SNP rs2910164 changes the expression of miR-146a, leading to an increased expression of its target genes YAP1 and COX2, potentially promoting cancer stem cell characteristics and increasing the risk of recurrence [95]. Similarly, the A allele of SNP rs2073859 disrupts the binding of miR-135a, reducing post-transcriptional regulation of LIMK2. This deregulation promotes enhanced cellular processes such as proliferation, migration, and invasion, and decreases apoptosis [96]. The lncRNA PCAT1 is influenced by SNP rs7463708 within its enhancer region. This SNP has been shown to enhance the binding of the androgen receptor transcription factor ONECUT2, increasing PCAT1 expression in the presence of androgens, which influence prostate cancer progression [97].

CNVs, structural variations in the genome resulting in abnormal numbers of DNA sections, can overlap with regions encoding ncRNAs, leading to changes in their expression and function [98]. In lung adenocarcinoma, for example, CNVs can amplify lncRNA ALAL-1, contributing to cancer cell proliferation and immune evasion [99]. Similarly, CNV also contributes to the oncogenic potential of PLANE by upregulating its expression, impacting cancer development and progression through its interaction with NCOR2 pre-mRNA splicing and hnRNPMM [100]. Furthermore, ncRNAs can influence DNA methylation targets, either activating or inhibiting them, and this regulation is also modulated by CNVs [101]. Specifically, lncRNAs can interact with DNA methyltransferases (DNMTs), recruiting or inhibiting these enzymes at chromatin loci, thereby affecting gene expression [102].

### 2.3 Elucidating ncRNA functions through single-cell

#### 2.3.1 Discovery and characterization of cell types

Single-cell RNA sequencing (scRNA-seq) is revolutionizing our understanding of cellular complexity. This advanced technique has uncovered a multitude of previously unrecognized cell types and states. In the field of neuroscience, for instance, Zeisel et al. utilized scRNA-seq to classify cell types in the mouse somatosensory cortex and hippocampus. Their study identified 47 distinct subclasses, including a variety of neurons and glial cells, each characterized by unique gene expression profiles [103]. This pivotal research expanded our catalog of neural cell types and shed light on their functional and developmental interconnections.

In immunology, Villani et al. applied scRNA-seq to dissect the human immune system, revealing an extensive array of immune cell types [104]. The team characterized new subtypes of dendritic cells, monocytes, and progenitors, each distinguished by distinct gene expression patterns. These discoveries have significant implications for understanding immune responses and the development of targeted immunotherapies.

In the oncology domain, Patel et al. employed scRNA-seq to explore the cellular makeup of glioblastoma, an aggressive form of brain cancer [105]. Their research identified a diverse range of cell types within tumors, including various malignant subpopulations and non-malignant cells, each contributing differently to tumor progression and therapy resistance. Single-cell analysis has been crucial in understanding the role of CD4 + T cells in antitumor immunity. Notably, patients responding to immunotherapy showed a marked enrichment of Th1-like CD4 + T cells, essential in mediating therapeutic outcomes [106]. Furthermore, single-cell studies have identified specific subsets of TAMss (TAMs) with distinct roles in cancer development. For example, TAMs expressing C1Q have been implicated in shaping the tumor's immune microenvironment, influencing T cell recruitment and regulation [107]. Such research provides a more detailed perspective of tumor heterogeneity, vital for developing effective personalized treatments.

In a related study, Gouin et al. combined scRNA-seq with spatial transcriptomics and proteomics to investigate the cellular details of bladder cancer[108]. They identified a novel subpopulation of epithelial cells characterized by CDH12 expression, which exhibits stem-like properties. This subgroup significantly affects the tumor's response to treatments, including surgery, chemotherapy, and immunotherapy. This study not only deepened our understanding of cellular heterogeneity in bladder cancer but also opened new avenues for targeted treatment strategies and personalized medicine. More findings on cellular heterogeneity are shown in Table 2.

**Table 2:** Single-cell sequencing reveals the heterogeneity of urological malignancies.

Tumor Type	Technology and Methods	Key Findings	Ref
RCC	scRNA-Seq	Identified a novel tumor-specific macrophage subpopulation prognostic for recurrence characterized by upregulation of C1Q, APOE, TREM2, and LILRB5	[109]
	scRNA-seq and ST	Nine distinct macrophage clusters were identified, each showing unique enrichment patterns at the tumor core and periphery	[110]
	scRNA-seq and TCR-seq	An enrichment of CD8A+ tissue-resident T cells has been associated with positive responses to ICB therapy. Conversely, an increased presence of TAMs has been observed in patients resistant to ICB	[111]
	scRNA-seq and scATAC-seq	Tumor cell-specific regulatory programs were identified, mediated by key transcription factors (TFs): HOXC5, VENTX, ISL1, and OTP	[112]
	scRNA-seq and scATAC-seq	Identification of specific long non-coding RNAs, RP11-661C8.2 and CTB-164N12.1, potentially contributing to the invasion and migration abilities of ccRCC	[113]
	scRNA-Seq	The expression of complement serine protease C1S in tumors correlated positively with levels of macrophage infiltration	[114]
	scRNA-Seq	Identification of SERPINE2 as a key gene for metastasis and EMT	[115]
BCa	scRNA-seq and TCR sequencing	Two cytotoxic phenotypes of CD4 + T cells, CD4GZMB and CD4GZMK, have been identified, capable of eliminating autologous tumor cells through an MHC class II-dependent pathway	[116]
	scRNA-seq	A new subpopulation of macrophages was identified in UCB, named Macro-C3. The Macro-C3 subset score is predictive of the response to immunotherapy in these patients	[117]
	scRNA-seq and ATAC-seq	Increased expression of EZH2 enhances stemness in cancer stem cells and diminishes cell adhesion in bladder cancer stem cells, thereby contributing to the recurrence of bladder cancer	[118]
BCa	scRNA-seq and TCR-seq	CD57 + CD8 T cells in the peripheral blood of mUC patients can serve as a potential biomarker for predicting patient response to atezolizumab	[119]
	scRNA-seq	Recruitment and activation of TAM by iCAF through CXCL12-CXCR4 as well as THY1, CSF signaling pathways plays a key role in orchestrating TME, which leads to T-cell depletion and establishes an immunosuppressive environment conducive to tumor progression	[120]
PCa	scRNA-seq	Four tumor endothelial cell subtypes have been identified: arterial, venous plexus, immature vascular, and tip cells. The CXCR4/CXCL12 axis, mainly expressed in arterial cells and associated with angiogenesis, presents a potential target for anti-angiogenic therapy in prostate cancer	[121]
	scRNA-seq	EEF2 + and FOLH1 + luminal subpopulations are exclusive to LNM in PCa. MYC facilitates PCa progression and TME immunosuppression by modulating PDL1 and CD47	[122]
	scRNA-seq	Primary prostate cancer typically shows a low presence of tumor-infiltrating immune cells, and mCSPC exhibits a robust immune infiltrate	[123]
	scRNA-seq	Cancer cell–derived IL-1β enhances MARCO expression in macrophages, which in turn fosters lipid uptake and promotes cancer cell migration through the release of CCL6 by lipid-loaded TAMs	[124]
	scRNA-seq	EP4 (PTGER4) is expressed in various PCa immune cells. The EP4 antagonist YY001 inhibits MDSC function, boosts T cell activity, and alters chemokine profiles, thereby reducing MDSC and T cell infiltration in the TME	[125]

#### 2.3.2 Multifaceted Roles of ncRNAs Revealed by scRNA-seq

scRNA-seq has been instrumental in uncovering the diverse and complex roles of ncRNAs in cellular processes. This technique offers a high-resolution analysis of gene expression at the individual cell level, enabling detailed observation of the nuanced regulatory functions of ncRNAs across development, health, and disease.

For example, scRNA-seq facilitated the identification of a lncRNA, lncMMPA, within exosomes from tumor-associated macrophages (TAMs), which plays a crucial role in the malignancy of hepatocellular carcinoma (HCC). The study revealed that lncMMPA promotes tumor cell glycolysis and proliferation by sequestering miR-548 s, leading to the upregulation of ALDH1A3. This discovery sheds light on the contribution of TAM-derived exosomes to HCC progression, positioning lncMMPA as a potential prognostic marker [126].

In the context of cancer research, scRNA-seq has been vital in exploring the role of ncRNAs in intratumoral heterogeneity and drug resistance. Zhao et al. found that the lncRNA NEAT1 was upregulated in specific metabolically advantaged subpopulations within hepatobiliary tumor organoids. Notably, in the CD44 positive cells of the HCC272 organoid, NEAT1 expression correlated with drug resistance mediated through the Jak-STAT pathway [127]. Additionally, scRNA-seq uncovered ZNFX1 antisense RNA 1 (ZFAS1) in sorafenib-resistant HCC cells. The increased expression of ZFAS1 is associated with enhanced stemness and EMT, contributing to a drug-resistant phenotype [128].

In cutaneous melanoma (SKCM), PRRT3-AS1, another lncRNA identified through scRNA-seq, was found to be highly expressed in advanced cases and associated with poor prognosis. This lncRNA promotes cancer cell migration, potentially through the EMT signaling pathway, and may influence immune cell infiltration, thereby affecting responses to immunotherapy [129].

Furthermore, scRNA-seq has been utilized to dissect the complex regulatory landscape of miRNAs during epithelial-to-mesenchymal transition (EMT). This research emphasized the role of the miR200 family in maintaining epithelial characteristics, highlighting their interaction with ZEB transcription factors. MiR101 was also noted for its role in preserving the epithelial phenotype by targeting EZH2, suggesting its potential as a therapeutic target 130].

These studies underscore the power of scRNA-seq in revealing the intricate functions of ncRNAs in regulating cell identity and their pivotal roles in disease processes, thereby opening new avenues for therapeutic intervention.

### 2.4 Deciphering lncRNA Functions: Computational Insights and Structural Revelations

The rapidly expanding field of lncRNAs is gaining significant attention for its potential in deciphering the regulatory architecture of the genome. LncRNAs, as critical modulators of gene expression and epigenetic regulation, are not only central to scientific exploration but also pivotal in identifying new therapeutic approaches.

LncRNAs deep involvement in vital cellular operations and disease pathways underscores the urgency of enhancing our comprehension of their functions. This complex landscape of lncRNA functionality calls for an interdisciplinary approach, combining traditional laboratory experimentation with computational innovation. Recent strides in computational biology have yielded advanced methods for identifying lncRNAs [217]. For example, deep learning-based tools like 'lncRNA-Mdeep' have shown exceptional efficacy in distinguishing lncRNAs from coding sequences, extracting intricate features from sequence data [131]. These tools have demonstrated superior performance in discriminating between lncRNAs and coding sequences by extracting complex features from sequence data [132], [133]. Similarly, 'lncRScan-SVM', employing SVM-based algorithms, has demonstrated notable accuracy in lncRNA identification, integrating both sequence and structural features and underscoring the importance of secondary structures in lncRNAs [134].

In terms of structural understanding, approaches like cross-linking and immunoprecipitation (CLIP) combined with HTS have illuminated lncRNA binding sites and interaction domains, revealing their spatial configurations and protein interactions [135], [136]. Additionally, the use of cryo-electron microscopy has been instrumental in revealing the three-dimensional structures of lncRNA complexes. A notable example is the elucidation of the compaction domain of the lncRNA XIST, which plays a crucial role in X-chromosome inactivation [137].

Computational predictions have also significantly enhanced our understanding of lncRNA functions. Tools like 'lncPath' use novel algorithms to predict lncRNA functions based on gene ontology term enrichment in co-expression networks, providing more precise insights into the roles of lncRNAs in biological pathways [138], [139]. 'LncADeep', integrating deep learning, has been crucial in revealing the regulatory roles of numerous uncharacterized lncRNAs, especially in cancer contexts [140]. DeepLncLoc, with its innovative approach to predicting lncRNA subcellular localization, has provided essential functional insights [141]. Lastly, the updated LncBook 2.0 enhances lncRNA annotations with multi-omics data, furthering research into their biological roles and associations with diseases [142].

These developments underscore the dynamic nature of computational and structural studies in elucidating lncRNA functions. As we continue to improve these technologies, our understanding of lncRNA roles in health and disease is set to expand significantly, bridging current knowledge gaps and opening new avenues for therapeutic interventions.

## 3 NcRNAs and their implications in urological malignancies

### 3.1 The complex roles of NcRNAs in bladder cancer pathogenesis

NcRNAs have emerged as crucial elements in our understanding of bladder cancer, playing vital roles from the initial stages of tumor formation to the disease's metastatic spread. Their significant impact on the progression of bladder cancer is detailed in Table 3.

**Table 3:** Influence of NcRNA on Proliferation, Metastasis, and Chemotherapy Resistance in urological malignancies.

Cancer Type	Non-coding RNA	Targets	Mechanism of Action	Biological Impact	Ref
Bladder Cancer	microRNA-381	BMI1 and the Rho/ROCK axis	miR-381 directly binds to BMI1 and negatively regulates its expression, leading to decreased RhoA phosphorylation and ROCK2 activation.	Upregulation of miR-381 can inhibit tumor cell proliferation, invasion, migration, anti-apoptosis and tumor formation	[149]
	microRNA-148b-3p	PTEN	CAF-derived exosomes transport miR-148b-3p into bladder cancer cells, targeting PTEN.	Downregulation of miR-148b-3p promotes PTEN expression and suppresses EMT, metastasis, and chemoresistance in bladder cancer cells.	[143]
	lncRNA-ZEB2NAT	TGFβ1	TGFβ1 induces epithelial-mesenchymal transition of bladder cancer cells through lncRNA-ZEB2NAT	Enhances cell proliferation, migration, invasion, and tumor growth to promote bladder cancer progression	[156]
	lncRNA-UCA1	Hypoxic exosomes	Hypoxic bladder cancer cells secrete lncRNA-UCA1-containing exosomes, which are then internalized by other bladder cancer cells.	Enhances cell proliferation, migration and invasion to promote bladder tumor growth and development	[155]
	lncRNA SNHG3	miR-515-5p/GINS2 axis	Upregulated SNHG3 positively regulates GINS2 expression through the sponge miR-515-5p under a ceRNA mechanism.	Promote bladder cancer proliferation, migration, invasion and EMT process	[154]
	lncRNA SNHG20	Wnt/β-catenin signalling pathway	Activation of the Wnt/β-catenin signaling pathway.	SNHG20 enhances cell survival, proliferation, colony formation, migration and invasion and inhibits apoptosis in tumors	[150]
	lncRNA SNHG14	microRNA-211-3p/ESM1 axis	SNHG14 up-regulates ESM1 via competitive binding with miR-211-3p.	Silencing of SNHG14 or elevation of miR-211-3p inhibited the tumorigenic ability of bladder cancer cells, including cell cycle entry, colony formation, invasion, migration, and proliferation abilities, and promoted cell apoptosis.	[144]
	lncRNA SNHG1	miR-143-3p and EZH2	SNHG1 enhances HK2 expression by sponging miR-143-3p. In the nucleus, SNHG1 interacts with EZH2 and regulates the histone methylation of the CDH1 promoter.	SNHG1 promotes tumor progression by enhancing cell proliferation, migration, invasion, and tumor growth and metastasis.	[151]
	lncRNA PVT1	Wnt/β-catenin signalling pathway	PVT1 is overexpressed in multidrug-resistant BUC tissues and cell lines, and its knockdown reduces BUC cell proliferation, invasiveness, and chemoresistance by modulating Wnt/β-catenin signaling.	PVT1 promotes bladder urothelial carcinoma progression by enhancing cell proliferation, invasiveness, and chemoresistance.	[152]
	lncRNA PSMA3-AS1	microRNA 214-5p/Programmed cell death-ligand 1 (PD-L1) axis	PSMA3-AS1, induced by YY1, functions as a competing endogenous RNA for miR-214-5p, regulating PD-L1.	PSMA3-AS1 promotes bladder cancer cell viability and metastasis, and inhibits apoptosis.	[145]
	Circular RNA_0000629	microRNA-1290/Cell Division Cycle 73 (CDC73) axis	circ_0000629 sponges miR-1290 and up-regulates CDC73 expression.	Knockdown of circ_0000629 inhibits tumor cell growth and metastasis	[148]
	circRNA_0000326	microRNA-338-3p/ETS Proto-Oncogene 1/phosphoinositide-3 kinase/Akt pathway	Circular RNA_0000326 sponges miR-338-3p and up-regulates ETS1 expression, activating the PI3K/AKT pathway.	Circular RNA_0000326 promotes bladder cancer cell growth and migration, and inhibits apoptosis.	[146]
	circLPAR1	miR-762	Regulation of invasion and metastasis through its interaction with miR-762.	Low expression of circLPAR1 is associated with enhanced invasion and metastasis of bladder cancer	[153]
	circCEP128	microRNA-515-5p/Syndecan-1 (SDC1) axis	circCEP128 sponges miR-515-5p and up-regulates SDC1 expression.	Knockdown of circCEP128 inhibits tumor cell growth and migration and promotes tumor cell apoptosis	[147]
Prostate Cancer	MicroRNA-99b-5p	mTOR/AR axis	miR-99b-5p targets and inhibits the mTOR/AR axis, inducing autophagy	Inhibit proliferation and induce apoptosis of prostate cancer cells, increase sensitivity to docetaxel	[157]
	miR-124-3p	EZH2	Directly targets and inhibits EZH2	Inhibits prostate cancer cell proliferation and invasion, and promotes apoptosis	[158]
	MiR‐129–5p	CAMK2N1	Binds specifically to the target mRNA sequence of CAMK2N1 and downregulates the expression of CAMK2N1	promotes docetaxel resistance	[159]
	miR-145-5p	Phospholipase D 5 (PLD5)	miR-145-5p directly targets and inhibits PLD5	Decrease in cell proliferation, invasion, and migration	[160]
	miR-221-5p	Not described	miR-221-5p overexpression reduces cancer cell migration	inhibit tumor growth	[161]
	LINC01963	miR-216b-5p/TrkB	Highly expressed LINC01963 promotes docetaxel chemotherapy resistance by regulating the miR-216b-5p/TrkB axis.	Silencing of LINC01963 enhances chemosensitivity to docetaxel	[162]
	lncRNA SNHG11	IGF-1 R, miR-184	upregulates IGF-1 R expression and sponges miR-184	Facilitates prostate cancer progression and metastasis.	[163]
	LOXL1-AS1	miR-let-7a-5p/EGFR	Upregulation of lncRNA LOXL1-AS1 promotes cell proliferation and migration while inhibiting apoptosis.	provides a potential therapeutic strategy for patients with drug-resistant prostate cancer.	[164]
	PCAT6	IGF2BP2 and IGF1R	Forms a complex with IGF2BP2 and IGF1R, stabilizing IGF1R mRNA and activating downstream pathways	promotes bone metastasis and tumor growth in prostate cancer	[165]
	PlncRNA-1	smad3	Sponge miR-136, PlncRNA-1 increases the expression of smad3	Enhance tumor cell proliferation, invasion and EMT	[166]
	CASC11	YBX1	CASC11 interacts with YBX1 to mediate the transcriptional repression of p53	Promote tumor progression and migration	[167]
	circHIPK3	miR-193a-3p and MCL1	Acts as a sponge for miR-193a-3p and upregulates the expression of the oncogene MCL1	Enhance tumor cell proliferation, migration and invasion	[168]
	Circ-XIAP	miR-1182/TPD52 Axis	Circ-XIAP sponges miR-1182, thereby regulating the expression of TPD52	the silencing of Circ-XIAP suppresses tumor growth and enhances docetaxel sensitivity	[169]
Renal Cell Carcinoma	SNHG12	CDCA3	SNHG12 upregulates CDCA3	Contributes to tumor progression and sunitinib resistance	[170]
	PCED1B-AS1	miR-484/ZEB1	PCED1B-AS1 upregulates ZEB1 by sponging miR-484	Promote cell proliferation, migration and EMT of tumor cells	[171]
	PCAT1	miR-656 and miR-539/YAP	PCAT1 upregulates YAP by sponging miR-656 and miR-539	Promote cell proliferation, migration and invasion of tumor cells	[172]
	PANDAR	Bcl-2 family proteins, PI3K/Akt/mTOR pathway	PANDAR promotes apoptosis by downregulating Bcl-2 and Mcl-1 and inhibiting PI3K/Akt/mTOR pathway.	Silencing of PANDAR suppresses tumor growth and promotes apoptosis	[173]
	lncRNA MALAT1	miR-362-3p and G3BP1	MALAT1 acts as a ceRNA for miR-362-3p, which controls the expression of G3BP1	Knockdown of MALAT1 inhibits tumor chemotherapy resistance, cell proliferation and invasiveness, and enhances apoptosis.	[174]
	LncARSR	miR-34 and miR-449/AXL and c-MET	lncARSR upregulates AXL and c-MET by sponging miR-34 and miR-449	Promotes resistance to sunitinib	[175]
	IGFL2-AS1	TP53INP2 pre-mRNA/hnRNPC	IGFL2-AS1 binds to hnRNPC proteins, regulates TP53INP2 and enhances autophagy.	Promote sunitinib resistance	[176]
	HOTAIR	miR-17-5p/Beclin1	HOTAIR upregulates Beclin1 and increases autophagy via sponge miR-17-5p	Promote sunitinib resistance	[177]
	HEIRCC	EMT-associated markers	HEIRCC activates EMT in a ZEB1-dependent manner	Promotes RCC cell spread	[178]
	Circular RNA Eps15	miR-4731-5p/ABCF2	Circular RNA Eps15 upregulates ABCF2 through sponge miR-4731-5p	Promote sunitinib resistance	[179]

The progression of bladder cancer is influenced by a myriad of molecular pathways, with diverse RNA types playing critical roles. A notable instance is the role of microRNA-148b-3p, derived from exosomes of cancer-associated fibroblasts (CAFS), which significantly inhibits tumor growth. This inhibition is achieved by suppressing the Wnt/β-catenin pathway and enhancing PTEN, both of which are key in tumor suppression[143]. Conversely, the lncRNA SNHG14 exacerbates bladder cancer progression by interacting with the microRNA-211–3p/ESM1 axis [144]. By sequestering microRNA-211–3p, SNHG14 induces an increase in ESM1, a protein that facilitates cell proliferation, migration, and invasion—essential processes in cancer development. Furthermore, the lncRNA PSMA3-AS1 acts as a competing endogenous RNA (ceRNA) for microRNA-214–5p, enhancing cell viability while reducing apoptosis, thereby contributing to tumor growth [145]. CircRNAs also play a role in the progression of bladder cancer. For example, circRNA-0000326 promotes disease progression by activating the phosphoinositide-3 kinase/Akt pathway through the sequestration of microRNA-338–3p, thus supporting cell survival and growth [146]. In contrast, the inhibition of circCEP128 and circRNA\_0000629 has been shown to impede bladder cancer progression, suggesting their potential as therapeutic targets [147], [148]. MicroRNA-381's involvement in bladder cancer progression is marked by its suppression of tumor growth and metastasis via downregulation of BMI1 and inactivation of the Rho/ROCK axis, critical pathways in cancer progression [149].

Bladder cancer invasion and metastasis, key determinants of prognosis and patient survival, are influenced by various ncRNAs. The lncRNA SNHG20, for instance, plays a notable role in advancing bladder cancer by activating the Wnt/β-catenin signaling pathway, a crucial driver of cell proliferation and tissue invasion [150]. This underlines the direct impact of ncRNAs on significant signaling pathways. Beyond influencing signaling pathways directly, ncRNAs contribute to bladder cancer progression through complex regulatory networks. For example, lncRNA SNHG1 promotes bladder cancer progression via interactions with miR-143–3p and the epigenetic modifier EZH2. This suggests a multi-faceted regulatory mechanism at play [151]. Additionally, the overexpression of lncRNA plasmacytoma variant translocation 1 (PVT1) in multidrug-resistant urothelial bladder carcinoma tissues and cell lines is linked to increased cell proliferation, invasion, and chemoresistance [152]. CircLPAR1, proposed as a biomarker for muscle-invasive bladder cancer, seems to regulate invasion and metastasis by interacting with miR-762, demonstrating the intricate interplay between ncRNAs and miRNAs in cancer progression [153]. The lncRNA small nucleolar RNA host gene 3 (SNHG3) further exemplifies this complexity, promoting bladder cancer proliferation and metastasis through the miR-515–5p/GINS2 axis [154]. LncRNA-urothelial cancer-associated 1 (lncRNA-UCA1), prevalent in hypoxic tumor-derived exosomes, has been implicated in promoting bladder tumor growth and development [155]. Lastly, lncRNA-ZEB2NAT, induced by TGF-$\beta$1 in CAFs, is involved in the epithelial-mesenchymal transition of bladder cancer cells, a crucial step in cancer metastasis [156].

The diverse functions of ncRNAs in bladder cancer highlight their potential as diagnostic biomarkers, prognostic indicators, and therapeutic targets.

### 3.2 Deciphering the multidimensional impact of NcRNAs in prostate cancer

NcRNA play an integral role in the complex process of carcinogenesis, steering the course from initiation to metastasis in prostate cancer. A plethora of evidence has identified numerous ncRNAs that are key players in directing these intricate processes within this disease [180], [181], [182].

For instance, microRNA-99b-5p in bladder cancer assumes multiple roles. It suppresses the expression of mTOR, a critical regulator of cellular growth and proliferation, thus inducing apoptosis and slowing cell proliferation. Additionally, this microRNA enhances the susceptibility of prostate cancer cell lines to the anti-cancer drug docetaxel, indicating its potential role in combating drug resistance. It also promotes autophagy, potentially leading to cell death through mTOR inactivation [157]. Similarly, miR-145–5p acts as a significant player by inhibiting Phospholipase D5 (PLD5), a protein known for promoting cell proliferation and metastasis. This leads to reduced cell proliferation, invasion, and migration, and it also hinders the expression of metastasis-associated proteins like N-cadherin, E-cadherin, Vimentin, and Snail, while blocking the AKT pathway, essential for cell survival and growth [160]. miR-124–3p specifically targets and reduces the expression of EZH2, a gene promoting cell proliferation and metastasis, thereby decreasing cellular proliferation, invasion, and migration [158]. Similarly, overexpression of miR-221–5p is associated with reduced cell proliferation and cancer cell migration [161]. Focusing on lncRNAs, PCAT6 has been identified as a key promoter of prostate cancer tumor growth, invasion, and metastasis. Its overexpression, particularly in prostate cancer with bone metastasis, correlates with worse prognosis. PCAT6 stabilizes the mRNA of IGF1R through its interaction with IGF2BP2 and IGF1R, activating pathways that encourage tumor growth and metastasis [165]. In a similar mechanism, CASC11 enhances prostate cancer development by interacting with YBX1 and repressing the tumor suppressor gene p53 [167]. Overexpression of lncRNAs PlncRNA-1 and SNHG11 in prostate cancer is also critical for cell proliferation and metastasis. Notably, SNHG11 boosts IGF-1R expression and recruits miR-184, leading to increased prostate cancer cell proliferation, invasion, and migration [163], [166]. The role of circRNAs is equally complex. CircHIPK3, for instance, functions as a sponge for miR-193a-3p, a tumor suppressor microRNA. Its upregulation in prostate cancer cells hinders miR-193a-3p’s ability to suppress the oncogene MCL1, thereby promoting cell survival and disease progression [168].

Chemoresistance poses a significant challenge in effectively treating prostate cancer. MiR-129–5p, for example, enhances resistance to docetaxel by modulating CAMK2N1 expression, which in turn promotes cell survival and aids cancer cell migration and invasion [159]. The lncRNA LOXL1-AS1, upregulated in doxorubicin-resistant prostate cancer DU-145 cells, increases cell proliferation and migration, while reducing apoptosis, implicating the LOXL1-AS1/miR-let-7a-5p/EGFR axis in drug resistance [164]. LINC01963 and Circ-XIAP have distinct roles in modifying prostate cancer cell sensitivity to docetaxel. LINC01963 counteracts miR-216b-5p's inhibition of TrkB, a protein known to promote drug resistance, potentially contributing to a chemoresistant phenotype. In contrast, Circ-XIAP sequesters miR-1182, preventing it from downregulating TPD52, another protein implicated in drug resistance [162], [169].

In conclusion, the diverse roles of ncRNAs in prostate cancer, from tumor growth to chemoresistance, underscore their importance in disease progression and highlight their potential as targets for novel therapeutic strategies. Further research is necessary to fully unravel the complex interplay of these ncRNAs in prostate cancer.

### 3.3 NcRNAs: unraveling their intricate roles in RCC

The approach of ncRNAs as significant participants in the molecular schema of genitourinary cancers has necessitated re-considering their roles in cancer progression [183]. The lncRNA PANDAR, for instance, is linked with poor prognosis in clear cell RCC (ccRCC), the most common RCC subtype. PANDAR interacts with transcription factors and regulatory proteins, altering the transcriptional landscape to promote tumorigenesis [173]. In contrast, the lncRNA HEIRCC is associated with metastatic behavior in RCC, facilitating the spread of RCC cells through its role in EMT, a crucial process in metastasis [178].

Recent studies have identified a hypoxia-associated lncRNA signature in ccRCC, including lncRNAs like SLC16A1-AS1, which are thought to modulate the cellular response to hypoxia, a hallmark of RCC, thereby influencing tumor growth and progression [184], [185]. The lncRNA PCAT1 is known to fuel ccRCC by enhancing the transcriptional coactivator YAP. It acts as a molecular sponge for miR-656 and miR-539, miRNAs that usually suppress YAP expression, illustrating lncRNAs' role as competing endogenous RNAs in RCC [172]. Similarly, lncRNA PCED1B-AS1 advances ccRCC progression through the miR-484/ZEB1 axis, hypothesized to sponge miR-484 and thereby alleviate the suppression of ZEB1, a key EMT inducer [171]. It is hypothesized to sponge miR-484, thus relieving the suppression of ZEB1, a prominent EMT inducer.

Chemoresistance in RCC adds further complexity to treatment, as it allows cancer cells to persist and resist elimination. The lncRNA lncARSR, for instance, induces resistance to sunitinib, a standard therapeutic agent for RCC. It acts as a ceRNA, sequestering miR-34 and miR-449, which would typically inhibit the AXL and c-MET genes associated with drug resistance and cancer progression [175]. Another lncRNA, SNHG12, promotes RCC progression and sunitinib resistance by upregulating CDCA3, a protein involved in cell cycle regulation, thus enhancing cancer cell proliferation and survival [170]. HOTAIR contributes to sunitinib resistance in RCC by acting as a ceRNA for miR-17–5p, thereby preventing the microRNA from suppressing Beclin1, a gene linked to autophagy, a process associated with drug resistance. This leads to increased autophagy, fortifying the cancer cells' resistance to sunitinib [177]. Furthermore, the lncRNA IGFL2-AS1 enhances TP53INP2 expression by binding competitively to hnRNPC, a protein that typically represses TP53INP2. The consequent upregulation of TP53INP2 promotes autophagy, leading to sunitinib resistance [176]. lncRNA MALAT1, overexpressed in sunitinib-resistant RCC tissues and cells, acts as a ceRNA for miR-362–3p, regulating the expression of G3BP1, a gene associated with chemotherapy resistance. Knockdown of MALAT1 in sunitinib-resistant RCC reduces chemotherapy resistance, cell proliferation, and invasiveness, while enhancing apoptosis [174]. Finally, the circRNA Eps15 hinders the action of miR-4731–5p, which usually suppresses ABCF2, a gene linked to drug resistance. By sponging miR-4731–5p, Eps15 increases ABCF2 expression, augmenting sunitinib resistance [179].

In conclusion, ncRNAs, particularly lncRNAs and circRNAs, are key to the development of chemoresistance in RCC. They offer potential targets for novel therapeutic strategies to overcome drug resistance and improve patient outcomes.

## 4 TME and its pivotal role in cancer progression

### 4.1 A comprehensive overview of the TME

**Fig. 1:**
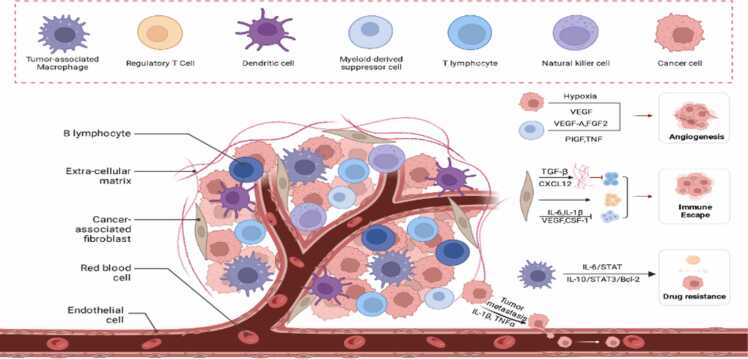
A Comprehensive Overview of the TME in urological malignancies.

The initiation and progression of cancer are significantly influenced by the TME, a complex and dynamic ecosystem comprising various cellular and non-cellular components. In a simplified view, the TME encompasses neoplastic cells, the extracellular matrix (ECM), immune cells, stromal cells, endothelial cells, and a range of signaling molecules (Fig. 1). These elements interact synergistically, establishing the TME as a crucial factor in cancer development [186]. Central to the TME are angiogenic vascular cells, essential in new blood vessel formation (angiogenesis), facilitating tumor nourishment and expansion. The role of immune cells within the TME is dualistic; while some attack the tumor, others may unintentionally aid its growth. CAFs often accelerate tumor progression by remodeling the ECM and releasing growth factors. Moreover, acellular components like the ECM and tumor vasculature are integral to the TME. The ECM provides structural support and acts as a growth factor reservoir, while architectural changes in the ECM can stimulate tumor progression [187], ]188]. The tumor vasculature, crucial for nutrient and oxygen supply, can create conditions like hypoxia and acidosis, promoting tumor growth and therapeutic resistance [189], ]190]. Furthermore, extracellular physicochemical conditions such as altered pH, hypoxia, and fibrosis significantly impact the TME, influencing tumor dynamics and interactions with the surrounding environment. This leads to metastasis, immunosuppression, and drug resistance [191].

Within the TME, the Tumor Immune Microenvironment (TIME) comprises various immune cells that significantly affect tumor behavior through their interactions with cancer cells and other TME constituents [192], [193]. T cells within the TIME can exert anti-tumor effects, whereas cancer cells may manipulate the TIME to evade immune surveillance by upregulating immune checkpoint proteins [193], [194]. TAMs within the TIME can promote tumor growth and metastasis by secreting growth factors and pro-angiogenic factors and producing immunosuppressive cytokines, thus impeding anti-tumor immune responses [195].

The interplay of each component within the TME is instrumental in dictating cancer progression. Understanding these interactions is vital for developing effective cancer therapies.

Cancer cells, as central players in the TME, profoundly impact cancer progression. They orchestrate complex interactions within the TME, aiding their survival, proliferation, and spread. These cells secrete substances such as growth factors, cytokines, and chemokines, stimulating angiogenesis to ensure a steady supply of nutrients and oxygen for tumor growth [196]. A prime example is the production of vascular endothelial growth factor (VEGF) by cancer cells, promoting endothelial cell proliferation and migration, leading to new blood vessel formation [197]. Additionally, cancer cells manipulate the immune landscape of the TME. They attract and activate immune cells like TAMs, MDSCs, and Tregs, fostering an immunosuppressive environment conducive to tumor growth and facilitating immune evasion [198]. For instance, cancer cells release Chemokine (C-C Motif) Ligand 2 (CCL2) to draw TAMs and MDSCs into the TME. These cells then produce anti-inflammatory cytokines such as IL-10 and TGF-β, suppressing the body's natural anti-tumor immune response [199]. Cancer cells also contribute to the formation of a fibrotic and hypoxic TME. They produce ECM components and activate Hypoxia-Inducible Factors (HIFs), creating a protective barrier against immune detection while triggering metabolic changes that promote tumor growth and treatment resistance [200]. Furthermore, cancer cells alter the function of stromal cells, such as CAFs and endothelial cells, through signaling molecule secretion. TGF-$\beta$, secreted by cancer cells, activates CAFs, leading to ECM component production and growth factor release, enhancing tumor growth and metastasis [201].

In conclusion, cancer cells play a pivotal role in modulating the TME, significantly influencing cancer progression.

### 4.2 Exploring the TME's Influence on Urological Malignancies

The intricate relationship between the TME and urological cancers, including prostate, bladder, and kidney cancers, is a critical focus of contemporary cancer research.

Within the TME, fibroblasts and endothelial cells play pivotal roles. They secrete various growth factors and cytokines that are instrumental in fostering the growth, survival, and metastasis of cancer cells. In bladder cancer, for instance, CAFs significantly contribute to disease progression by releasing factors such as the Hepatocyte Growth Factor (HGF) and VEGF, which aid in tumor development and spread [202].

Immune cells within the TME also significantly influence the progression of urological malignancies. Tumor-infiltrating lymphocytes (TILs), comprising both CD8 + and CD4 + T cells, possess the capacity to target and eliminate cancer cells. However, in many cases of urological cancers, the effectiveness of these immune cells is diminished due to the immunosuppressive nature of the TME. For example, RCC typically harbors immunosuppressive cells like regulatory T cells (Tregs) and Myeloid-Derived Suppressor Cells (MDSCs), which hinder the activity of cytotoxic T cells and promote tumor progression [203].

Moreover, alterations in the ECM) within the TME, such as increased collagen deposition and crosslinking, play a significant role in cancer progression. In prostate cancer, these ECM modifications lead to a stiffer tumor structure [204]. As suggested by Chevrier et al. [203], such changes may promote cancer cell invasion and metastasis by supporting cancer cell survival, proliferation, and immune evasion. Therefore, a comprehensive understanding of the TME's multifaceted influence on urological cancer progression is crucial.

The TME of urological cancers is a complex ecosystem comprising tumor cells, extracellular matrix, a variety of immune cells, stromal cells, and endothelial cells. These components interact through signaling molecules such as HIF-1α, Vascular Endothelial Growth Factor, IL-6, and IL-10, influencing tumor progression, immune evasion, and response to treatment in urological cancers. The figure was created with BioRender.com.

## 5 Elucidating NcRNA and the TME interactions in urological malignancies

**Fig. 2:**
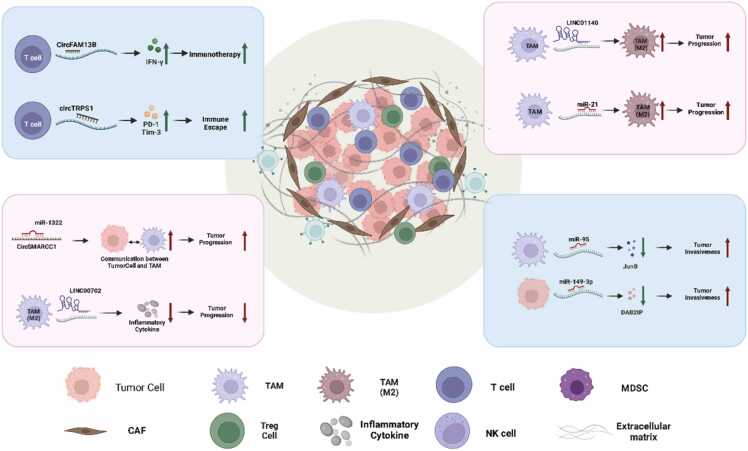
The interaction of ncRNA with the TME in Urological Malignancies.

Recent advances in cancer research have highlighted the significant role of ncRNAs in urological cancers such as prostate, bladder, and kidney cancers, particularly in the context of their interaction with the TME. The interplay between ncRNAs and the TME is crucial in cancer onset, development, and treatment resistance (Figure2, Table 4).

**Table 4:** Impact of ncRNA-TME Interactions on Malignancy in Urological Malignancies.

Cancer Type	Non-coding RNA	Tumor Microenvironment	Biological function	Ref
Bladder Cancer	miR-21	M2 Macrophages	miR-21 activates the PI3K/AKT pathway in macrophages and enhances M2 macrophage polarization	[209]
	LINC00702	M2-TAMs	Inhibition of bladder cancer cell proliferation and secretion of inflammatory cytokines by M2-TAM	[206]
	LINC01140	M2 Macrophages	Affects bladder cancer cell aggressiveness and macrophage M2 polarization, promoting cancer progression	[208]
	circFAM13B	CD8 + T cells	Overexpression of circFAM13B can cause CD8 + T cells to secrete more IFN-γ, thereby increasing the immunotherapy sensitivity of bladder cancer cells.	[205]
	circTRPS1	T Cells	circTRPS1 can induce T cell exhaustion in TME	[210]
Renal Cell Carcinoma	miRNA-21-5p	M2 Macrophages	Exosomes from M2 macrophages containing miRNA-21-5p regulate PTEN/AKT signaling to promote RCC metastasis	[211]
	microRNA-155	Hypoxic Conditions	Inhibits apoptosis and promotes migration and proliferation of RCC cells by targeting FOXO3 expression	[212]
Prostate Cancer	CircSMARCC1	TAMs	Promotes PCa progression by disrupting the crosstalk between tumor cells and TAMs via miR-1322/CCL20/CCR6 signaling	[213]
	miR-149-3p	Inflammatory Factors	Activates NF-κB and enhances TNF-dependent induction of pro-invasive inflammatory factors, promoting cancer progression and metastasis	[214]
	miR-95	Tumor-Associated Macrophages	Promotes PCa progression via exosome-mediated transfer	[215]
	miR-423-5p	CAFs	Promotes chemotherapy resistance by targeting GREM2 through the TGF-β signaling pathway	[216]

### 5.1 NcRNA-TME interactions in bladder cancer

The TME of bladder cancer showcases dynamic interactions between cancer cells and immune cells, notably involving circRNAs and lncRNAs.circFAM13B, for instance, overexpressed in bladder cancer, stimulates CD8 + T cells to secrete IFN-γ, enhancing immunotherapy effectiveness.It also interferes with IGF2BP1's binding to PKM2, reducing glycolysis in cancer cells [205]. Another key molecule, LINC00702, inhibits bladder cancer cell growth and inflammatory TME development by recruiting JUND to promote DUSP1 gene transcription, essential for halting cancer progression. This lncRNA also reduces the release of inflammatory cytokines from M2-TAMs, which are known for aiding bladder cancer progression [206]. Additionally, LINC01140, associated with muscle-invasive bladder cancer, correlates with FGF9 and promotes M2 macrophage polarization, facilitating cancer cell invasiveness and progression[207], [208]. In addition, ncRNAs also influence macrophage polarization and T cell exhaustion in the TME. For example, miR-21, found in exosomes from bladder cancer cells, impacts macrophage polarization by downregulating PTEN and activating the PI3K/AKT pathway, leading to enhanced M2 polarization and cancer progression [209]. circTRPS1 in exosomes modulates T cell exhaustion by affecting PD-1 and Tim-3 expression on CD8 + T cells, contributing to immune evasion and tumor advancement [210].

### 5.2 NcRNA-TME interactions in RCC

In RCC, the TME presents a unique landscape where ncRNAs significantly affect cancer progression. miRNA-21–5p, encapsulated in exosomes released by M2 macrophages, promotes RCC metastasis by modulating the PTEN/AKT signaling pathway, essential for cancer cell survival and spread [211]. In addition to miRNA-21–5p, miR-155 transported within extracellular vesicles (EVs) in the TME enhances RCC cell viability and contributes to tumor progression. Hypoxic conditions in the TME increase the release of these EVs, with miR-155 targeting FOXO3, a protein crucial for cell cycle regulation and apoptosis, thus accelerating RCC cell proliferation, invasion, and metastasis[212].

### 5.3 NcRNA-TME interactions in prostate cancer

In prostate cancer, the interplay between ncRNAs and the TME is critical for understanding disease progression and identifying potential therapeutic targets. CircRNA CircSMARCC1 influences the TME by modulating chemokine secretion. It binds to miR-1322, enhancing CCL20 secretion, which interacts with CCR6 on TAMs, facilitating tumor cell and TAM communication that promotes tumor progression [213]. Another significant player is miR-149–3p, which directly impacts cancer cells and the surrounding TME. By downregulating the tumor suppressor DAB2IP, it activates NF-κB, a key effector in cancer cell invasion. This modification not only escalates the aggressiveness of cancer cells but also influences the cells' response to inflammatory cytokines present in the TME, fostering a pro-invasive environment [214]. Furthermore, miR-95 plays a crucial role in the cross-talk between TAMs and cancer cells. Transferred via exosomes from TAMs to cancer cells, miR-95 downregulates JunB, a transcription factor essential for cell proliferation and apoptosis. This transfer promotes cancer cell proliferation, migration, and invasion, underlining the complex interplay within the prostate cancer TME [215]. Lastly, the role of miR-423–5p, secreted by CAFs in exosomes, is noteworthy. Upon transfer to prostate cancer cells, it induces chemoresistance by targeting and downregulating GREM2 via the TGF-β signaling pathway. This interaction significantly affects the efficacy of chemotherapy drugs like docetaxel, taxane, and bicalutamide, presenting a challenging obstacle in treatment [216].

The complex interactions of ncRNAs within the TME of urological cancers underscore their potential as therapeutic targets. While research has illuminated their role in tumor progression, metastasis, and drug resistance, a deeper understanding of these intricate relationships is necessary for developing novel treatments.

This figure illustrates the complex interaction between ncRNAs and the TME in urological malignancies. It presents how specific ncRNAs affect cellular behavior and tumor progression by influencing immune responses, cell viability, migration and invasiveness within the TME. It highlights the pivotal role of ncRNAs in tumor development and their potential as therapeutic targets. The figure was created with BioRender.com.

## 6 Conclusion

This review article provides a comprehensive of the biogenesis and regulatory mechanisms of ncRNAs, with a particular focus on their varied roles within the TME. We delve into the complex interactions between ncRNAs and urological malignancies, unveiling their significant potential in cancer diagnosis and treatment.

Recent advances in HTS technologies and bioinformatics have substantially enriched our understanding of ncRNAs, elucidating their functions and intricate interactions within the TME. There is a growing body of evidence highlighting the crucial involvement of ncRNAs in the etiology of urological cancers. The application of ncRNAs in diagnostic and therapeutic strategies offers promising prospects for enhancing clinical outcomes. However, research into the complex relationship between ncRNAs and the TME in urological malignancies is still in its initial stages. A more expansive understanding of ncRNA's role in cancer progression and tumor development is needed. Currently, the majority of research focuses on single or limited numbers of ncRNAs, which restricts a complete grasp of their roles in cancer processes and the tumor environment in urological systems.

For ncRNAs to be effectively used in clinical settings for the diagnosis and treatment of urological malignancies, further experimental validation and methodological development are essential.

## Author contributions

GW provided the theme and conceived the manuscript's structure. SW, XQ and DL participated in the initial research and wrote the manuscript. DX, BJ and JW guarantee the accuracy and completeness of the manuscript. XW reviewed and revised the manuscript. All authors contributed to the manuscript and approved the submitted version.

## CRediT authorship contribution statement

**Shijin Wang**: Writing – review & editing, Visualization. **Xiaochen Qi**: Writing – original draft preparation. **Dequan Liu**: Writing – original draft preparation. **Deqian Xie**: Investigation. **Bowen Jiang:** Investigation. **Jin Wang**: Investigation. **Xiaoxi Wang**: Methodology, Supervision. **Guangzhen Wu:** Conceptualization, Project administration, Funding acquisition.

## Declaration of Competing Interest

I affirm that the present work is original, has not been previously published, and is not currently under consideration for publication elsewhere. Additionally, there are no conflicts of interest associated with this study.
